# Fibrinogen and mucin binding activity of EF0737, a novel protein of *Enterococcus faecalis*

**Published:** 2017-12

**Authors:** Samira Sabzi, Rahil Mashhadi, Mohammad Reza Pourmand

**Affiliations:** 1 Department of Pathobiology, School of Public Health and Biotechnology Research Center, Tehran University of Medical Sciences, Tehran, Iran; 2 Urology Research Center, Tehran University of Medical Sciences, Tehran, Iran

**Keywords:** *Enterococcus faecalis*, EF0737, Fibrinogen, Mucin, Pathogenesis

## Abstract

**Background and Objectives:**

*Enterococcus faecalis* is the leading cause of several human infections. This opportunist pathogen expresses surface components that have various functions in the infection process including bacterial adhesion, lytic activity, and induction of host immune responses. EF0737, a novel cell wall associated protein, may play an important role in pathogenesis of *E. faecalis*, based on our experiments. This study was conducted to clone and express EF0737 and demonstrate its interaction with biotinylated plasma proteins and patients’ sera.

**Materials and Methods:**

The full length of *ef0737* gene was cloned in pTZ57R/T cloning vector and subcloned in *p*ET21a expression vector. Recombinant protein expressed in *Escherichia coli* Origami (DE3) was confirmed by western blot technique, using anti-His tagged monoclonal antibodies, and was then purified. Interaction of the recombinant protein with plasma proteins and patients’ sera were examined by western blot.

**Results:**

The *ef0737* gene was successfully cloned and expressed in *E. coli* Origami host. Binding activity was observed between the purified EF0737 recombinant protein and fibrinogen and mucin among other plasma proteins. Moreover, reaction was also observed between the purified product and sera obtained from patients diagnosed with *E. faecalis* infection.

**Conclusion:**

The observed reactions between EF0737 and fibrinogen, mucin and patients’ sera suggest that EF0737 may play important role in pathogenesis of infections caused by *E. faecalis*. However, more comprehensive characterization of this novel protein may provide better understanding of host pathogen interaction.

## INTRODUCTION

During the last decades, enterococci have been recognized as a major cause of nosocomial infections worldwide. Among the species of this genus, *E. faecalis* accounts for about 80% of all enterococcal infections (1). It is one of the leading causes of urinary tract infections, surgical wound infections, bacteremia, and 5% to 15% of all bacterial endocarditis (2). The treatment of these diseases has become challenging due to the development of multidrug resistance of *E. faecalis* and absence of novel antibiotics. Deeper knowledge of the pathogenicity of *E. faecalis* may go a long way in bridging the gaps in treatment and prevention of *E. faecalis* infections (3).

Although knowledge on the virulence factors of *E. faecalis* is still limited, several pathogenic determinants including cytolysin, aggregation substance, extracellular superoxide, and surface proteins have been described in *E. faecalis* (4). In particular, surface protein components interact with the human extracellular matrix (ECM) or immobilized plasma proteins, and play a fundamental role in colonization, and thus contribute to bacterial pathogenicity (5). Because of the key role of surface proteins in the host-pathogen interaction, they are interesting targets for drug and vaccine design. To achieve this, a deeper insight of adhesin determinants and their antigenicity properties is required (6).

Prior studies have demonstrated that *E. faecalis* can bind different parts of ECM, such as collagen, laminin, fibronectin, fibrinogen, and lactoferrin, but the entity responsible for these adhesions are still not well-distinguished. Recently, some studies have reported the importance of surface proteins as considerable adhesins to ECM components in *E. faecalis* (7, 8).

In the present study, we described molecular characterization of EF0737, a novel protein encoded by *ef*0737 gene. EF0737 is an enzyme protein with amidase role in *E. faecalis* (9). Bacterial amidase has multi-functions, such as autolytic activity, cell-division, and bacterial attachment. These activities might help microorganisms to persist and survive in the host. A part from *E. faecalis* in other Gram-positive cocci, such as *Staphylococcus aureus* and *Streptococcus pneumoniae*, some roles of autolysin have been well-demonstrated. An autolysin of *S. aureus* (Aaa) is bifunctional and has both enzymatic (amidase and glucosaminidase) and adhesive functions; also, it mediates binding to fibrinogen and fibronectin (10). Similar functions were found in *S. pneumoniae* (11).

In this study, we focused on the binding activity and antigenicity properties of EF0737 protein. To investigate EF0737, the *ef*0737 gene was cloned, overexpressed, and purified. The binding ability of EF0737 to plasma proteins and antigenicity properties to the sera of patients with *E. faecalis* infection were examined. In our previous study, we demonstrated that *ef*0737 gene is conserved in different strains of *E. faecalis* and can be used to detect clinical *E. faecalis* isolates.

## MATERIALS AND METHODS

### Bacterial strains, plasmids and culture media.

*E. faecalis* ATCC29212, containing full length *ef*0737 gene, was obtained from Urology Research Center of Sina Hospital, Iran. *E. coli* strain DH5α was used as the host for recombinant plasmid. Moreover, *E. coli* Origami B (DE3) was used as expression host. Furthermore, pTZ57R/T (Thermo Fisher Scientific, US) as T/A cloning vector and *p*ET21a (Novagen, USA) as expression vector were used in experiments. *p*ET21a is a bacterial vector with the size of 5.4kb. LB agar and broth were used for culturing *E. coli* strains.

### DNA extraction and PCR amplification of *ef*0737 gene.

Chromosomal DNA of *E. faecalis* was extracted using DNA Extraction Kit (Bioneer, Seoul, South Korea), based on the manufacturer’s instructions and was used as template for PCR amplification. The upstream (5′-GCGCGCCATATGTCTAAATTTTTAAAAGTAATCGG-3′) and the downstream (5′-CGCGCGCTCGAGCTGCTCATCTCTATTTATTTTTTTA-3′) primers (20pmol/μL) with the underlined restriction sites were used to obtain a 1587-bp product. A high fidelity PCR reaction was set with the following thermal cycles: 5 minutes at 95°C for one cycle, 1 minute 30 cycles at 95°C, 45 seconds at 63°C, 90 seconds at 72°C and a final extension cycle of 5 minutes at 72°C.

### Cloning of *ef*0737 gene.

The resulting PCR product was tailed by an oligo A at 3′ side and was subsequently cloned into the pTZ57R/T vector, using T4 Ligase enzyme (Takara, Japan), generating pTZ-*ef*0737. Then, the recombinant vector was transformed into *E. coli* DH5α. Restriction mapping and bidirectional sequencing of cloned fragment was performed to confirm the construct. To prepare the final construct, *ef*0737 cloned fragment was cut from pTZ-*ef*0737 vector using *Nde*I and *Xho*I enzymes (Fermentas, Germany) and subsequently cloned into *p*ET21a. Then, positive clones were selected from the ampicillin-supplemented LB agar (1μg/mL) plates and were confirmed by colony PCR, restriction analysis and sequencing.

### Expression of recombinant EF0737 and protein purification.

Competent cell was prepared using standard calcium chloride method and transformed to the expression host, *E. coli* Origami. Transformed cells were cultured on LB agar containing tetracycline (1/5μg/mL) and ampicillin (1μg/mL). For expression experiments, transformants were cultured in 5 mL LB broth and induced by adding IPTG (Fermentas, USA) 1mM/mL at the optical density of 0.4–0.6 in 600 nm. The bacteria were incubated by vigorous shaking for 2 and 4 hours at room temperature. Expression of EF0737 was analyzed by SDS-polyacrylamide gel electrophoresis (SDS-PAGE). For protein purification, *E. coli* Origami (DE3), containing *p*ET21a-*ef*0737 plasmid, was grown in large scale in LB broth, containing ampicillin (1μg/mL) and tetracycline (1/5μg/mL), and the pellets of bacterial cells expressing protein were centrifuged at 10 000 rpm for 5 minutes. The pellets were resuspeneded in binding buffer (8 M urea, 0.1 M NaH_2_PO_4_, and 0.01 M Tris, pH = 8.0) and centrifuged for 30 minutes at 12 000 rpm. Supernatant was added to Ni-NTA His-Bind*Resin gel (Sigma-Aldrich, US) and shaken for 1 hour at RT. Then, it was transferred to the column that contained glass wool at the bottom. The column was washed with denaturation wash buffer (8 M urea, 0.1 M NaH_2_PO_4_, and 0.01 M Tris, pH = 6.3) and renaturation wash buffers with descending molarity of urea. Finally, recombinant protein was eluted with elution buffer (50 mM NaH_2_PO_4_, 300 mM NaCl and 250 mM imidazole pH = 8.0) and analyzed by SDS-polyacrylamide gel-electrophoresis (SDS-PAGE).

### Confirm recombinant EF0737 protein by western blotting.

At first, western blot was used to confirm the His-tagged recombinant protein. Briefly, the recombinant protein was size-separated on SDS-PAGE gel. Then, it was electroblotted on nitrocellulose membrane. The membrane was blocked overnight (4°C) with 3% skimmed milk in PBS. Subsequently, it was washed 3 times in a washing buffer (Tween 20 and PBS), incubated in His-tag antibody (Sigma, USA), and diluted in 1:1000 in PBS for 1 hour at room temperature. Afterwards, the membrane was washed 3 times, and DAB substrate (3, 3′-Diaminobenzidine) (Sigma, USA) was added to detect the recombinant protein.

### Plasma proteins binding to EF0737 protein.

Plasma proteins binding was assayed by western blotting, using biotinylated human serum proteins, including fibrinogen, fibronectin, lactoferrin and mucin (12). The recombinant protein was electroblotted on nitrocellulose membrane as previously mentioned. After blocking and washing the membrane, it was incubated in plasma proteins diluted in 1:1000 for 3 hours at room temperature. Then, the membrane was washed as before, incubated in Streptavidin (Roche, Germany), and diluted in washing buffer in 1:30000 at room temperature for 3 hours. Subsequently, the membrane was washed and incubated in alkaline phosphatase buffer for 5 minutes. Then, the membrane was immersed in NBT/BCIP (Roche, Germany) solution and kept in dark condition until the protein band appeared. The ScaB recombinant protein was used as negative control.

### Reactivity of serum antibody with recombinant EF0737 protein.

To investigate whether EF0737 is expressed in human body during infection, we screened sera from hospitalized patients infected with *E. faecalis* for serological response against EF0737 by western blotting. Serum samples were collected from 7 different patients diagnosed with *E. faecalis* infection at Shariati Hospital affiliated to Tehran University of Medical Sciences (2016–2017). Recombinant EF0737 protein transferred to nitro-cellulose membrane. After blocking with blocking buffer overnight (4°C), the membrane was washed with washing buffer containing 0.05 Tween 20 and incubated with sera diluted in 1:1000 for 2 hours at room temperature. Then, the membrane was washed 3 times with washing buffer. After wash step, goat anti-human Ig peroxidase-conjugated (Cyto matin Gene Co, Isfahan, Iran) with a dilution of 1:30000 was added and incubated for 2 hours at room temperature. Finally, NBT/BCIP (Roche, Germany) was added as substrate, and the reaction was stopped after 5 minutes. Two serum samples obtained from healthy individuals were used as negative controls.

## RESULTS

### Cloning of *ef*0737 gene.

**Fig. 1.**
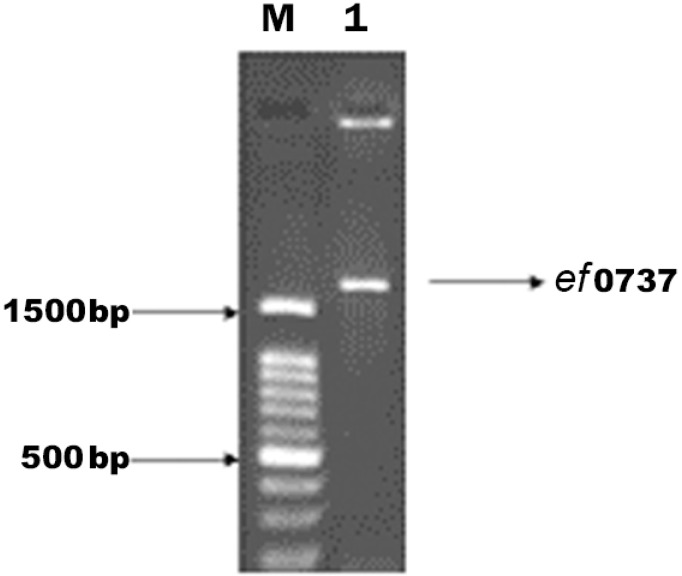
Agarose gel electrophoresis of the amplified *ef*0737 gene by PCR

**Fig. 2.**
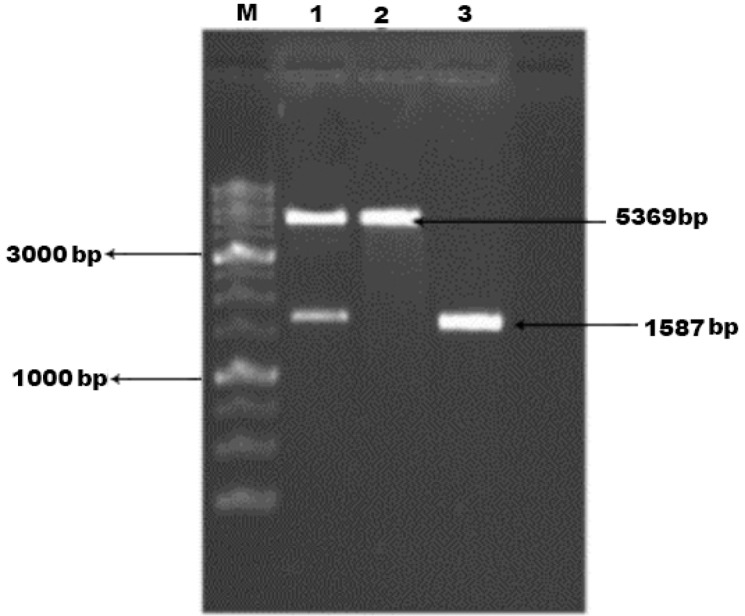
Electrophoresis of digested recombinant vector and PCR product on agarose gel (1%) M: DNA Marker (1Kb); Lane 1: Double digest of recombinant vector (*p*ET21a) with *Xho*I and *Nde*I; Lane 2: Digested *p*ET21a Vectors with *Xho*I and *Nde*I restriction enzymes, respectively; Lane 3: *ef*0737 PCR product (1587bp)

Using the specific primers, the *ef*0737 gene of *E. faecalis* was amplified, showing a band about 1600 bp on agarose gel (Fig. 1). The purified PCR product (1587bp fragment) was first subcloned into pTZ57R/T vector, then, it was cloned into the *p*ET21a plasmid for the expression of the EF0737 protein. The accuracy of cloned target was confirmed after double digestion with *Nde*I and *Xho*I enzymes and sequencing stages. The result of double digestion is presented in Fig. 2.

### Expression of *ef*0737 gene and purification of recombinant protein.

**Fig. 3.**
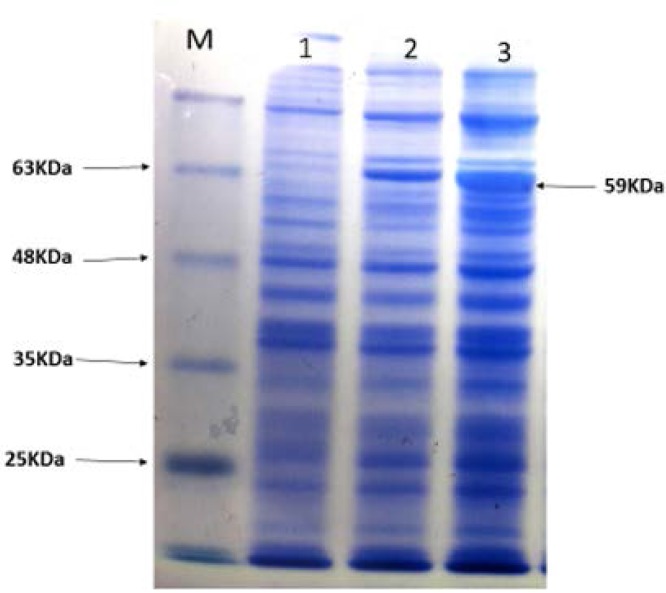
Identification and analysis of the recombinant protein by SDS-PAGE (12.5%) Lane M: Protein size marker (Sinaclon, Iran); Lane 1: Whole bacteria without induction; Lane 2: Whole bacteria 2 hours after induction; and Lane 3: Whole bacteria 4 hours after induction

**Fig. 4.**
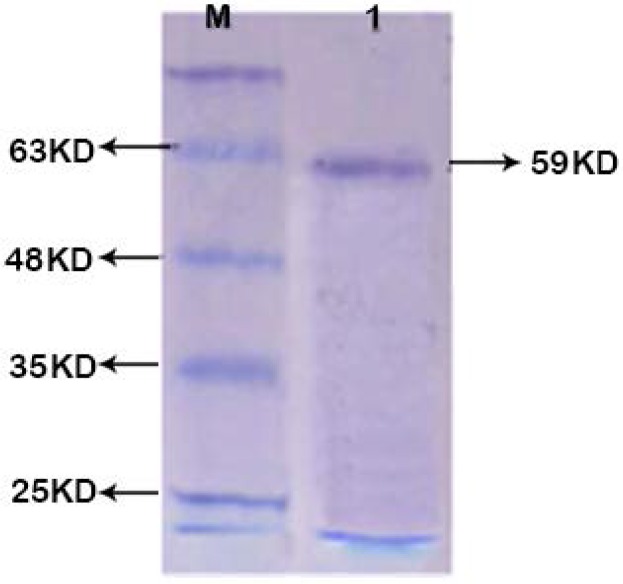
SDS-PAGE analysis of purified recombinant protein Lane M: Protein size marker (Sinaclon, Iran); lane1: Elution stage of EF0737 protein

**Fig. 5.**
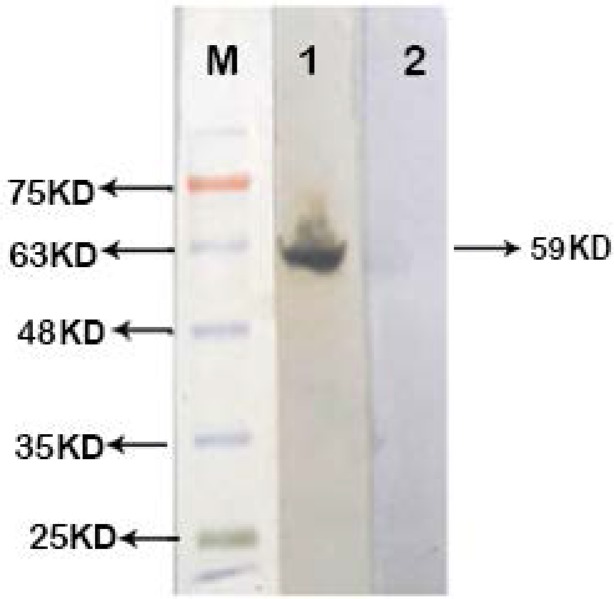
Western blot analysis of purified recombinant protein EF0737 Lane M: prestained protein size marker (Sinaclon, Iran); Lane 1: Recombinant protein western blotting with His-Tag monoclonal antibody; lane2: Negative control (*E. coli* without recombinant protein)

As described above, recombinant protein was successfully expressed in *E. coli* Origami (DE3) expression host (Fig. 3), and the insoluble protein. The best expression condition was obtained at 4 hours at room temperature after IPTG addition. The recombinant protein was purified using Ni-NTA His-Bind*Resin gel (Fig. 4). The His-tagged recombinant EF0737 protein was confirmed using anti-His-antibody (Fig. 5).

### Interaction of recombinant EF0737 protein with fibrinogen and mucin.

**Fig. 6.**
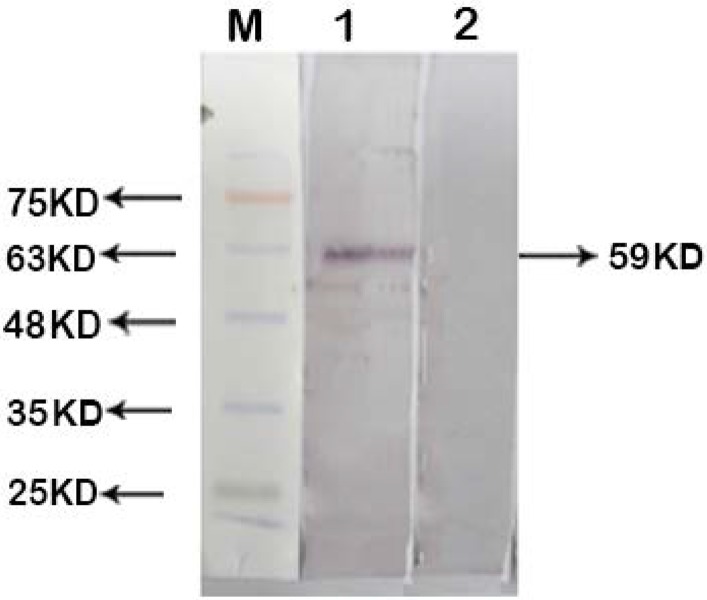
Analysis of interaction between EF0737 and fibrinogen protein. Lane M: Prestained protein size marker (Sinaclon, Iran); Lane1: EF0737 has a positive interaction with fibrinogen; Lane 2: Negative Control (ScaB recombinant protein)

**Fig. 7.**
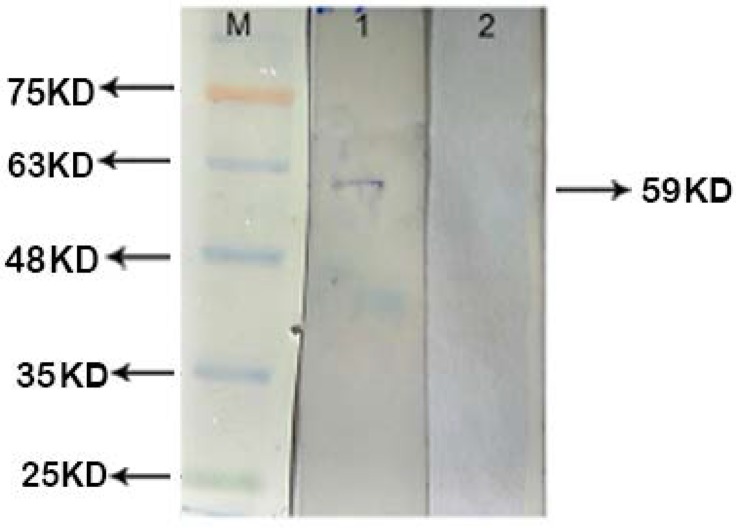
Analysis of interaction between EF0737 and mucin protein Lane M: Prestained protein size marker (Sinaclon, Iran); Lane1: EF0737 has a positive interaction with mucin; lane 2: Negative control (ScaB recombinant protein)

Interaction of recombinant EF0737 protein with plasma proteins was evaluated using western blot, and the results revealed that the recombinant protein has a positive reaction with fibrinogen and mucin proteins (Figs. 6, 7). Previously, the efficiency of plasma proteins was investigated by specific antigens binding to them.

### Reactivity of recombinant EF0737 protein with serum antibodies from enterococcal patients.

**Fig. 8.**
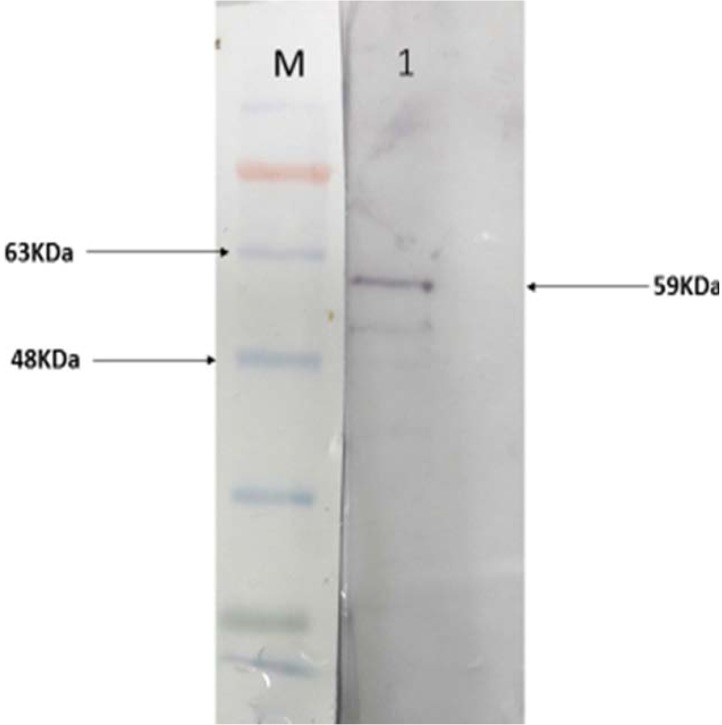
Antigenicity activity of EF0737 protein. Lane M: pre-stained protein size marker (Sinaclon, Iran); lane1: reaction of EF0737 with serum antibody of patient

Using western blot, we demonstrated that EF0737 protein has antigenicity activity and reacted to patients’ sera (Fig. 8).

## DISCUSSION

In general, knowledge about colonization and pathogenic mechanisms by *E. faecalis* is limited, and this is in contrast with what is known for some Gram-positive pathogens, such as *S. aureus*; virulence elements including toxins or adhesion factors are well-known (13). Most previous studies investigated the binding of the *E. faecalis* isolates to soluble or immobilized ECMs, while the binding markers have not been well-studied (14).

EF0737 is a novel unique protein in *E. faecalis* that contributes to several physiological functions. Silico analysis of EF0737 with 528 amino acid sequence shows a putative N-terminal anchor membrane protein which has one transmembrane helix and is embedded in the hydrophobic region of the cell membrane (15). Although amidase activity is the main distinctive role of this protein enzyme (9), this study focused mainly on the ability of EF0737 to bind plasma proteins, such as fibrinogen and mucin and on demonstrating the reactivity of the recombinant protein to the sera obtained from patients diagnosed with enterococcal infection.

We were able to find that EF0737 protein interacts with fibrinogen and mucin. These results were consistent with other publication, which characterized surface proteins in various pathogens (16–18). Fibrinogen (Fg) is a heterogeneous dimeric glycoprotein, which plays a key role in hemostatic processes and participates in both innate and extrinsic host immune system. Thus, many pathogenic microorganism can bind to Fg and manipulate its biologic activity (19).

In a recent study, one of the considerable roles of surface protein Ebp (endocarditis-and biofilm-associated pilus) in *E. faecalis* infections process was elucidated. EfbA, which is a minor subunit at the tip of Ebp, mediates adherence to components of ECM, such as collagen, and average levels of binding to mucin, and fibrinogen (20).

In our study, EF0737 showed binding property to mucin. Mucin is the major building block of the mucus gel and is important in keeping up the mucus barrier for host-microorganisms interaction and bacterial adhesion (21). *Streptococcus pyogenes* M protein interacts with mucin of respiratory mucosal tissue (21). Also, bacterial pathogens, such as *Pseudomonas aeruginosa, Pseudomonas cepacia, S. aureus* and *Haemophilus influenzae* bind to mucin; however, the adhesion parts involved in this interaction have not been well-identified (22). Moreover, only a few studies have been conducted on mucin binding proteins in *E. faecalis*, despite the same loads of record in other Gram-positive bacteria.

In the present study, we demonstrated that EF0737 reacted with sera from hospitalized patients with *E. feacalis* infections, indicating that EF0737 is expressed during infection and is being recognized by human immune system. In conclusion, a comprehensive study on *E. faecalis* surface antigens, such as EF0737, may contribute to the development of novel intervention strategies for both therapeutic and prophylactic approaches in preventing enterococcal infections.
